# Digital health interventions in palliative care: a systematic meta-review

**DOI:** 10.1038/s41746-021-00430-7

**Published:** 2021-04-06

**Authors:** Anne M. Finucane, Hannah O’Donnell, Jean Lugton, Tilly Gibson-Watt, Connie Swenson, Claudia Pagliari

**Affiliations:** 1 Clinical Psychology, School of Health in Social Science, University of Edinburgh, Edinburgh, Scotland UK; 2 Marie Curie Hospice Edinburgh, Edinburgh, Scotland UK; 3 The Usher Institute, University of Edinburgh, Edinburgh, Scotland UK; 4 Edinburgh Medical School, University of Edinburgh, Edinburgh, Scotland UK

**Keywords:** Quality of life, Palliative care

## Abstract

Digital health interventions (DHIs) have the potential to improve the accessibility and effectiveness of palliative care but heterogeneity amongst existing systematic reviews presents a challenge for evidence synthesis. This meta-review applied a structured search of ten databases from 2006 to 2020, revealing 21 relevant systematic reviews, encompassing 332 publications. Interventions delivered via videoconferencing (17%), electronic healthcare records (16%) and phone (13%) were most frequently described in studies within reviews. DHIs were typically used in palliative care for education (20%), symptom management (15%), decision-making (13%), information provision or management (13%) and communication (9%). Across all reviews, mostly positive impacts were reported on education, information sharing, decision-making, communication and costs. Impacts on quality of life and physical and psychological symptoms were inconclusive. Applying AMSTAR 2 criteria, most reviews were judged as low quality as they lacked a protocol or did not consider risk of bias, so findings need to be interpreted with caution.

## Introduction

The diagnosis of a life-limiting illness, along with its management during periods of wellness, illness, remission, decline and end of life can be stressful for patients, caregivers and healthcare professionals. Palliative care offers a holistic set of approaches for ameliorating the physical, psychological, social and spiritual burdens that patients and their families can face when dealing with the challenges associated with advanced progressive incurable illness, end of life and bereavement^1,2^. It prevents and relieves suffering through early identification, assessment and symptom management; including addressing practical needs and providing bereavement support. Digital health interventions (DHIs) in palliative care need to address the holistic needs and preferences of people with deteriorating health; and maintain patient–professional relationships that are dignity-enhancing and focused on patient and caregiver values and goals^3^. Improving access to, and increasing the quality of palliative care delivered is a healthcare priority in many countries^4,5^. DHIs could have an essential role to play in achieving these aims.

Digital health, or eHealth, is a broad term used to refer to the application of information and communication technologies (ICTs) and networks for the management, delivery and optimisation of patient care and health services, and for supporting patients themselves. It encompasses a range of related concepts such as telemedicine and telehealth, mobile health (mHealth), health informatics and wearable devices^6,7^. The adoption of digital health technologies is rapidly changing how healthcare is provided. Electronic health records (EHRs) and decision support tools are part of routine healthcare practice in many countries, while the use of videoconferencing to provide care at a distance is becoming more common. Mobile phones, apps, wearables and social media are in widespread use by citizens/patients, and innovations such as augmented reality, virtual assistants and artificial intelligence (AI) are finding new uses in clinical management and patient self-care. These approaches are reshaping healthcare as they become more affordable and widespread^3^.

Palliative care is one area where these technologies are increasingly being deployed^8^. Research to establish the feasibility of using videoconferencing in palliative care was first reported 20 years ago^9^. In healthcare organisations, pathways and preferences for palliative care are being steadily integrated into EHRs^10^. In parallel, mobile applications and online social networks for supporting patients’ physical, cognitive and emotional needs are becoming popular, both supplied by healthcare providers^11^ and driven by patients and caregivers themselves^12^. More recently, predictive analytics and AI are being used to adapt clinical interventions to stages of terminal illness^13^.

Reflecting this activity, there has been a significant rise in the number of systematic reviews focused on DHIs and palliative care over the past 15 years^14–18^. Despite their general support for these approaches, the clinical scope and quality of existing reviews varies widely, making it difficult to evaluate their implications for the field as a whole. Given the growing demand for palliative care services worldwide^19^ and the increasing penetration of DHIs in healthcare, the time is right for a comprehensive synthesis and appraisal of this evidence base. We employed the meta-review method to capture, appraise and synthesise the evidence represented in the systematic review literature on DHIs in palliative care. Our objectives were:

1. To identify the DHIs used in context of palliative care described in existing systematic reviews.
2. To describe the overall quality of existing systematic reviews.
3. To synthesize evidence on the role and the effects of DHIs in palliative care.

## Results

### Search results

**Fig. 1:**
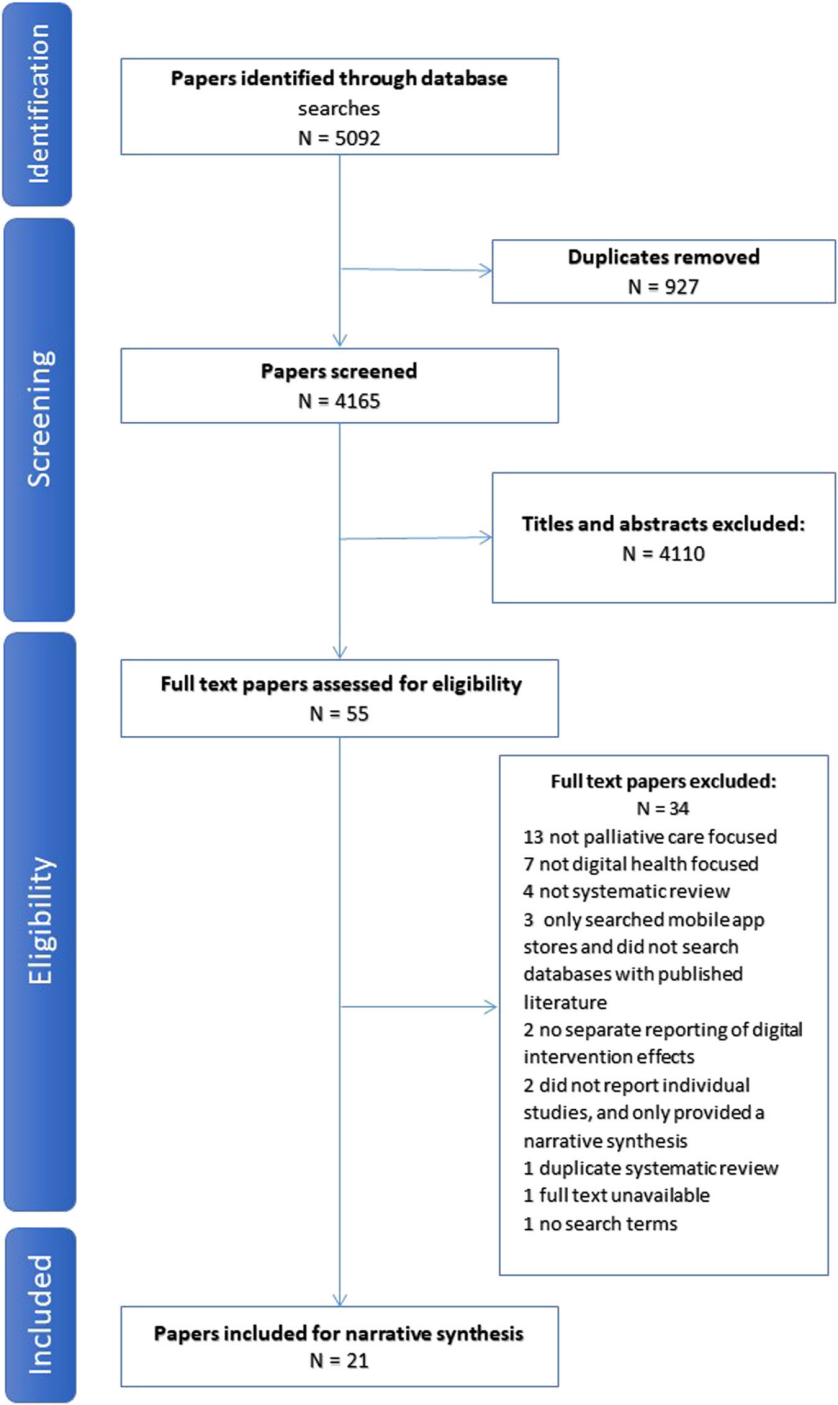
Prisma flow diagram. Overview of the search process.

The database searches returned a total of 5092 titles and abstracts, of which 55 potentially relevant papers were subjected to full-text review and 21 were eligible for inclusion (Fig. 1). The main reason for excluding articles at full-text review was that they were not focused on palliative care (13 studies) or DHIs (7 studies); not systematic reviews (4 studies); did not search databases of published literature (3 studies); did not report on effects of DHIs or provide detail on included reviews (4 studies) or other reasons (3 studies). During the search process, we identified one meta-review of telemedicine in palliative care published in 2016^20^. This meta-review identified a total of 6 systematic reviews published between 2007 and 2012, all of which were included amongst the 21 eligible reviews in this meta-review.

### Description of the included systematic reviews

Characteristics of the 21 included reviews are shown in Supplementary Table 1. Most included a range of study populations—patients, family members, caregivers and health professionals. Two reviews solely considered evidence on interventions for caregivers^18,21^, two were concerned with perspectives of healthcare professionals^22,23^, while one was applied specifically to paediatric palliative care^14^. Two reviews focused on cancer^24,25^, others did not limit their inclusion criteria to a specific disease. The reviews were carried out by research teams based on the following countries: USA (*n* = 9)^17,18,21,22,26–30^, UK (*n* = 6)^10,16,25,31–33^, Australia (*n* = 2)^14,34^, Canada (*n* = 1)^24^, Chile (*n* = 1)^15^, Denmark (*n* = 1)^35^ and Brazil (*n* = 1)^23^.

The 21 reviews were published between 2007 and 2019 and included primary research papers spanning 1997–2018. Ten systematic reviews covered broad areas such as telehealth^14,16,18,21,30,33^, telehospice^17^, ehealth^15^ and ICTs^25,26^. Eleven reviews had a more specific focus: EHRs^10,28,29,34^, internet^23,27^, weblogs^31^, mhealth^32^, telephone^24^, videoconferencing^35^ and simulators^22^.

**Fig. 2:**
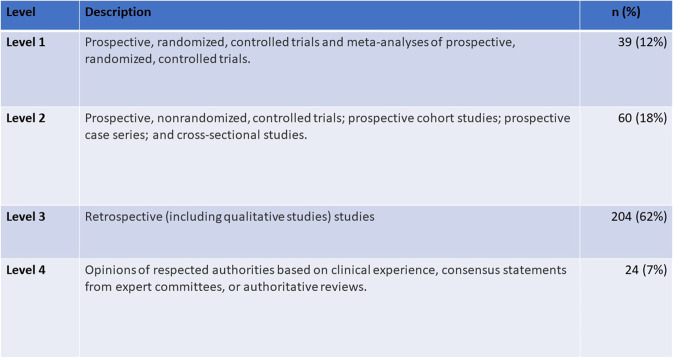
Publications categorised by PDQ Levels of Evidence. Excludes four systematic reviews and one PhD thesis.

The number of studies related to DHIs and palliative care in each review ranged from 5^32^ to 39^35^. Taken together the reviews summarised evidence from 332 unique publications, including four systematic reviews and one PhD thesis. Drawing on the Physician Data Query (PDQ) Levels of Evidence, used by the National Cancer Institute^36^ to appraise Supportive and Palliative Care studies, we categorised publications within the 21 systematic reviews into one of four levels. Level 1 represented study designs typically considered to offer the strongest evidence and Level 4 the weakest. Only 12% of publications were Level 1. Of the 43 publications describing randomised controlled trials (RCTs), 29 were unspecified trials, 13 were pilot or feasibility trials and one was a Phase II trial. Four of these were classed as Level 2 due to their scale or scope. Most publications described retrospective and qualitative designs (Level 3) (Fig. 2).

Most primary publications within the systematic reviews were included in only one review (70%, *n* = 279); one-fifth appeared in two publications (21%, *n* = 84), 7.5% appeared in three reviews (*n* = 30) and only 1.3% appeared in five reviews (*n* = 5). For further detail on the individual publications within each systematic review, see Supplementary Tables 1, 2 and Supplementary Data 1.

### Range of DHIs for palliative care described in existing systematic reviews and individual publications within the reviews

We classified the types of DHIs described in the 328 studies represented within the 21 reviews (Table 1). The most common types of DHIs involved videoconferencing or videophone (*n* = 56, 17%), EHRs (*n* = 51, 16%) and telephone or mobile phone (*n* = 41, 13%). Online interventions, including educational websites and online courses, were described in 31 publications (9%). Only six publications were focused on social media (2%), e.g. interactive online blogs. We found a relatively large proportion of publications describing mixed or unspecified DHIs (*n* = 50, 15%). Some DHIs were delivered using a mix of technologies or contained multiple components (e.g. telephone call with follow-up video-consultation).

**Table 1:** Types of DHIs reported in publications included in 21 systematic reviews.

Type of Digital Health Intervention	No. of publications	% of publications
Videoconferencing/videophone	56	17%
Electronic health records	51	16%
Phone/mobile phone	41	13%
Online	31	9%
Video	27	8%
Computer	20	6%
PDA/tablet/smartphone	16	5%
High-fidelity simulator	16	5%
Other (e.g. digital pens)	7	2%
Social media	6	2%
Telemonitoring	5	2%
Text messaging	2	1%
Mixed/unspecified	50	15%
Total	328	100%

DHIs were used for a range of purposes in palliative care (Table 2). A fifth of publications described DHIs for educational purposes (*n* = 64) most frequently involving online learning, simulators and videoconferencing targeting professionals. Symptom management was the main aim of DHIs outlined in 15% of publications (*n* = 49), and all types of DHI were used for this purpose. Information provision or management, often using EHRs, was the main aim of DHIs in 13% of publications (*n* = 44). Communication was the main aim of DHIs in 9% of publications (*n* = 29), with videoconferencing most often used. Decision-making support for patients and professionals was the main purpose of DHIs described in 13% of publications (*n* = 42)—video aids and EHRs were often used for this purpose. Overall, 15% of publications (*n* = 49) described DHIs for mixed or unspecified purposes. Mixed purposes could include information support and decision-making or communication and information sharing. Unspecified purposes had no specific focus.

**Table 2:** Main purpose of DHIs reported in publications included in 21 reviews.

Purpose of Digital Health Intervention	No. of publications	% of publications
Education	64	20%
Symptom management	49	15%
Information provision or management	44	13%
Decision-making support	42	13%
Communication	29	9%
Caregiver support	14	4%
Increase access to palliative care	11	3%
Out of hours care/emergency admissions	9	3%
Clinical follow-up	9	3%
Psychological or psychosocial support	8	2%
Mixed/unspecified	49	15%
Total	328	100%

### Quality of evidence

Applying the AMSTAR 2 critical appraisal tool for systematic reviews, only one review was judged as moderate quality^35^. At least one of the AMSTAR 2 ‘critical domains’ was missing from all other reviews (*n* = 20) (Supplementary Table 3), and overall quality of all other reviews was rated low (*n* = 15) or very low (*n* = 5) because of this. Only three systematic reviews referred to a study protocol or specific guide developed prior to the conduct of the review^22,33,35^. Most (*n* = 13) did not consider risk of bias. All were judged to have partially conducted a comprehensive literature review, though none had searched all sources identified in the AMSTAR 2 constituting a fully comprehensive search. Most reviews provided a satisfactory explanation for the heterogeneity of findings in their discussion (*n* = 17). Of the non-critical domains, none of the reviews explicitly defined all components of PICO when describing the research question, few provided a list of excluded studies (*n* = 3) and only one-third used a satisfactory technique for analysing risk of bias in individual studies. Most conducted study selection and data extraction in duplicate, reported sources of funding and potential conflicts of interest. Meta-analysis was not conducted in any review due to the heterogeneity of included study designs and outcomes.

Eleven systematic reviews assessed the quality of evidence of included publications^10,14,17,18,21,22,26,30,33–35^. Four used the Cochrane risk of bias tool^18,26,30,34^. One used the Critical Appraisal Skills programme tool^14^. Five reviews used different tools previously described in the literature^10,21,22,33,35^ while one developed a quality appraisal framework specifically for their review^17^. Three reviews described evidence as moderate-to-high quality^14,17,21^. Eight reviews reported evidence of low-to-moderate quality^10,18,22,26,30,33–35^. This was due to small sample size, insufficient detail on study design, unclear or high risk of bias, non-blinding of participants and outcomes, and poorly defined comparison groups.

### Role and effects of DHIs for palliative care

Findings from each review are described in relation to seven thematic areas: education, symptom management, information sharing, decision-making, communication, quality of life (QoL) and cost-effectiveness.

### Education

Eight reviews identified DHIs for education, of which most focused on describing interventions rather than evaluating their outcomes^15,16,21–23,26,32,33^. Educational interventions were delivered via online learning for professionals^15,18,23,26^, videoconferencing for professionals^16,33,37^, videos for professionals^19,26,29^, online symptom reporting for caregivers^21^, simulation-based learning experiences for professionals^22^ and mobile phones/text messaging for education and training of providers and patients^32^. Two reviews reported that online learning was a feasible alternative to in-person training, though quality of evidence of primary studies within these reviews was not assessed^16,23^. In a review of distance learning for healthcare professionals, Taroco et al. suggested that online case consultations involving active participation of students facilitated knowledge retention^23^. They also noted the prevalence of mixed educational initiatives (i.e., distance learning and classroom-based), with 64% of studies involving mixed approaches, suggesting a need for classroom activity to consolidate knowledge acquired at a distance. There was no consensus about the most-effective learning methods, and most virtual learning environments used a variety of multimedia to support communication and feedback mechanisms. Kidd et al.^16^ suggested that online learning and remote access to guidelines supports dissemination of good practice but also reported that face-to-face teaching methods are preferred when discussing emotional or psychological issues. Ostherr et al.^26^ reported strong evidence for benefits of video for educating patients about their illness and helping to determine treatment choices. Smith et al. sought to examine evidence on the use of simulation-based learning for end-of-life care conversations in their review, but found that information on outcomes was absent^22^. Overall, evidence on the impact of DHIs on education was mainly positive, though studies were mostly descriptive, outcomes assessed were heterogeneous, and evidence quality was not generally examined.

### Symptom management

Thirteen reviews referenced the role of DHIs in monitoring, assessing and managing physical and psychological symptoms^14–18,21,24–26,28,30,33,35^. EHRs were used to record symptoms^28,33^ while telephone and videoconferencing were frequently used to monitor, assess and treat symptoms^15,18,24–26,31,35^. Some reviews described positive impacts of DHIs on symptom management, while most reviews identified inconsistent evidence or noted that evaluation of impact in many studies was lacking. Describing evidence with moderate certainty, Jess et al.^35^ identified positive impacts of videoconferencing on symptom burden, especially in remote settings, though also noted negative impacts in some studies, specifically due to technical challenges, which caused communication problems. Zhou et al.^24^ concluded that telephone follow-up, for patients with advanced cancer, is a feasible alternative to hospital follow-up for symptom palliation and reduces travel burden. Head et al.^30^ reported positive or no impacts of DHIs on patient symptoms (e.g. physical and social functioning), noting that overall evidence from primary studies within their review was weak. Hancock et al.^33^ described home telemonitoring initiatives for patients (e.g. use of the telephone or computer software to record clinical symptoms at home); however, most interventions had not been evaluated. The heterogeneity of outcomes used to assess particular symptoms such as pain was highlighted by Allsop et al.^25^. Bush et al.^28^ described evidence linking the documentation of clinical symptoms on an EHR to reduced time in hospital in the last 6 months of life, though this finding was based on just one publication included in their review, and was not directly evaluated in others.

Seven reviews reported mostly positive effects of DHIs on psychological symptoms—anxiety, depression and distress^14,17,18,21,30,31,35^. Jess et al.^35^ identified largely positive impacts of videoconferencing on patient and caregiver anxiety, with the exception of one RCT, which found negative impacts^38^. This RCT compared weekly video-consultations by a palliative care specialist with treatment as usual in home-dwelling patients with advanced cancer. The RCT authors concluded that higher distress in the video-consultation arm may have been due to excess focus on symptoms and suffering, and the provision of pre-scheduled support over 3 months as opposed to when it was actually needed^38^. Bradford et al.^14^ described a number of small studies examining videoconferencing interventions for paediatric palliative care, noting reductions in anxiety. Head et al.^30^ identified positive effects of DHIs (telemonitoring and videoconferencing) on patient anxiety, depression and distress. Zheng et al.^18^ reported significant improvements in caregiver anxiety associated with access to videophones. Parker-Oliver et al.^17^ identified studies examining the effect of DHIs on anxiety, though studies were not large enough to detect significant differences in outcomes. Ngwenya et al.^31^ focusing on online blogging, reported that patients experienced a sense of emotional support, social connections and empowerment through writing online blogs.

### Information sharing

Eight reviews considered the information-sharing value of DHIs, with most describing the value of the information rather than evaluating specific outcomes^10,15,16,25,27–29,34^. In an early review of internet use, Willis et al. described the positive impacts of the internet as an additional source of information for patients, families and clinicians^27^. They found that patients and caregivers used online support groups and chatrooms to exchange information about an illness and alternative treatments. Patients and caregivers developed a connection with others online and appreciated the anonymity associated with online support. Capurro et al.^15^ reported that DHIs were used by clinicians, patients and caregivers to meet informational needs regarding pain and symptom management and medication use. Kidd et al. highlighted the importance of telephone helplines for general practitioners, nurses and caregivers for gathering information about managing symptoms and medical equipment^16^. These telehealth interventions improved the reliability and accuracy of information exchanged^16^. Allsop et al.^25^ noted that many systems designed to capture information from a patient for use by a healthcare professional, involved relaying symptoms without engaging in active forms of communication.

Four reviews highlighted the information-sharing function of EHRs in palliative care^10,28,29,34^. These reviews concluded that EHRs available across settings and platforms allow patient preferences regarding advance care planning (ACP) to be shared, improving continuity of care and ensuring that patients are treated in line with their wishes. Bush et al.^28^ reported that in low-resource settings, the implementation of a standalone EHR system capturing patient demographics and palliative care treatment information was found to significantly improve clinical workflow. Leniz et al.^10^ found that those with an EHR shared across settings were more likely to die in their preferred place compared with those who did not have an EHR. However, EHRs were limited in their capacity to capture important qualitative information such as information on anxiety or family distress^28^. Furthermore, locating relevant ACP information within the EHR was often challenging^34^, though could be improved by ensuring all ACP information is documented in a specific area^28^. Documentation templates, order sets and prompts may also improve the quality and incidence of ACP within EHRs^29^. Having an EHR improves documentation of advance care plans and communication of care planning information^28,29,34^, but this can come at the cost of increased workload^10^, challenges identifying which patients should have a shared EHR^34^, and concerns regarding data-sharing, security and consent^10^. Huber et al.^29^ suggest that further research focused on developing a consensus definition for ACP documentation and related quality elements in EHRs is needed.

### Decision-making

Four reviews considered the role of DHIs in decision-making by patients^26^ and professionals^23,28,34^. Ostherr et al. identified 20 studies where video, computer-based multimedia and online materials were used to support communication between patients, families and staff in context of end-of-life decision-making^26^. There was evidence for the efficacy of video in facilitating ACP decisions, resulting in improvements in completion of advance directives, discussion of end-of-life preferences and improved patient knowledge and satisfaction. Taroco et al. identified two studies on distance-learning courses for decision-making in palliative care, but did not describe the outcomes^23^. Two reviews considered the role of clinical decision support systems (CDS), including EHRs in facilitating decision-making^28,34^. Bush et al.^28^ described evidence on the use of such systems to identify patients for a palliative care approach, and to capture ACP directives and patient-reported outcomes to inform clinical decision-making. Due to heterogeneity of studies, evidence could not be synthesized. However, Bush et al.^28^ described positive impacts including a reduced likelihood of ICU admissions and hospital death for those with patient-reported outcomes shared via EHR, compared to those without; and earlier identification of patients for ACP discussion. Lemon et al.^34^ found that EHRs can improve documentation of advance directives. Electronic reminders, electronic templates, decision aids and standard locations of advance directives increase documentation. Electronic search systems and identification algorithms located within the EHR can assist with identification of patients who could potentially benefit from a palliative care approach, by flagging those who may have palliative care needs for review by the clinician. Overall, the evidence from publications included in Lemon et al. was weak, but points towards promising potential effects of EHRs for ACP.

### Communication

Ten reviews described the role of DHIs to facilitate communication between patients, professionals and caregivers using phones, internet and computer systems^15,21,22,25–27,30–32,35^. Positive effects included enhanced communication between patients, healthcare professionals and caregivers;^15,21,27,35^, more opportunities to express feelings^31^, increased connectednesss^15^, caregiver support^17^ and improved ACP^26^. Jess et al.^35^ identified 16 studies relating to the impact of videoconferencing on communication in palliative care. Positive impacts included greater efficiency and access, whereby several participants could be visually present and participate at once; shared decision-making involving the multidisciplinary team, patient and family; and enhanced communication through access to non-verbal as well as verbal responses. Negative impacts could occur where the family felt overwhelmed by the involvement of too many participants. Smith et al.^22^ found that simulation-based learning was frequently used to teach nursing students communication skills in palliative care settings, but due to the lack of standardization and poor evaluation, it was difficult to identify best practices.

### Quality of life (QoL)

Seven reviews considered the effects of DHIs on QoL^14,17,18,21,24,30,35^. Most reviews described improvements that were not statistically significant or positive impacts. Negative impacts were rarely observed. In their review of videoconferencing, Jess et al. identified several studies incorporating a QoL measure in their design, but QoL outcomes were not described in their findings^35^. Zheng et al. found no significant difference in QoL outcomes after telehealth interventions for caregivers^18^. Head et al. identified one study reporting a positive impact of telephone monitoring on QoL whereas another involving videophones showed no difference^30^. Similarly, in their review of telehealth for paediatric palliative care, Bradford et al.^14^ found either positive effects on QoL or no significant differences. Zhou et al. reported that telephone follow-ups with patients with advanced cancer reduced the patient burden by eliminating the need to come into hospital, facilitating a better QoL, though quality of evidence was not assessed and insufficient data on included studies was provided^24^. In a review of telehealth and hospice care, Oliver et al.^17^ reported that studies examining QoL were too small to identify clinically significant differences. In a review of weblogs in palliative care, Ngwenya and Mills^31^ concluded that weblogs improve patient and QoL by empowering patients and giving them a sense of active participation in their treatment, but this was a small scale study with no quality assessment of included studies. In reviews of EHRs, outcomes relating to QoL were rarely assessed^10,33^.

### Costs and resource use

Five reviews considered the financial implications of DHIs, with most reporting positive impacts of DHIs on costs for patients, caregivers or providers^14–17,35^. Jess et al. described cost savings associated with video consultation in palliative care for clinicians, service providers, patients and caregivers^35^. In two studies within their review, video consultations between healthcare professionals and patients resulted in cost savings for the hospital, compared to in-person consultations, and in clinician travel expenses for home visits. Travel cost savings were also noted for patients and carers in rural settings^35^. In a review of DHIs in hospices, Oliver et al.^17^ identified one telehospice cost analysis study; this study reported reduced costs for telehospice visits versus traditional hospice homecare. Bradford et al.^14^ described cost efficiencies when video visits were used in place of home visits; and when videoconferencing was used to educate patients about self-care, but cautioned that the cost-effectiveness will depend on whether DHIs are used in parallel with, or as a replacement for, traditional approaches. Kidd et al. described DHIs as an efficient alternative for patients and clinicians when time and distance is limiting^16^. Capurro et al. described cost efficiencies related to reduced hospital visits, but this was based on only one study in their review^15^. Overall, evidence on costs and resource use was positive, though interventions and outcomes assessed were heterogeneous, findings were based on a small number of primary studies within a small number of reviews, evidence quality was not generally assessed and robust economic evaluation not undertaken.

## Discussion

### Main findings

This meta-review indicates that DHIs in palliative care are being used for education, symptom management, information sharing, decision-making and communication, with the aim of improving patients’ QoL and the reach and efficiency of services. Positive impacts of DHIs were reported on education, information sharing, decision-making and communication in palliative care contexts. Evidence pertaining to physical and psychological symptoms and QoL was inconclusive or absent. No evidence of risks to patient safety was reported. However, the methodological quality of existing systematic reviews on DHIs for palliative care was low when judged using the AMSTAR 2 appraisal criteria, mainly due to the absence of review protocols and risk of bias assessment. DHIs can play a positive, enabling role in palliative care but there is a need for more rigorous evaluation, implementation and cost-effectiveness studies, with a greater focus on patient perspectives and consideration of bias in study designs. Rigorous quality guidelines should be adopted before embarking on future systematic reviews of primary research in palliative care to increase the confidence that can be placed in their findings.

### Advantages of this study

To date this is the most comprehensive meta-review focused on DHIs in palliative care. Compared to a previous meta-review, which encompassed six reviews^20^, it examined a wider range of databases and identified 21 systematic reviews for critical appraisal and synthesis. This meta-review shows that DHIs are more prevalent in palliative care than previously described, are used for a broader range of purposes, that impacts are generallyoften positive; and that the overall quality of research evidence is improving. As a by-product of our review, we provide a database of all 332 publications categorised by DHI type and main use, which may be of use to other researchers interested in evidence on specific DHIs for palliative care (Supplementary Data 1).

### Limitations of this study

The heterogeneity of review aims, methods and presentation of results created challenges for evidence synthesis. In many reviews, DHIs were described but outcomes were not evaluated in any detail. Although the searches were completed in January 2020, the dates of the primary studies ranged from 1997 to 2018, reflecting the time lag in academic publishing. None of the eligible systematic reviews focused on smartphone applications for palliative care, despite their growing use in this context^39,40^. Three reviews emerged after our searches had been completed, including a rapid review on video consultations in palliative care in context of COVID-19^41^, a scoping review of patient experiences of telehealth for palliative care at home^42^ and an integrative review of patient experiences of ehealth in palliative care^43^. Two would not have been eligible, as they were not systematic reviews. However, we suggest that future meta-reviews include all review types. Only 3 of the 21 systematic reviews had pre-registered a protocol on PROSPERO, which, in part accounts for the low-quality judgements obtained when using the AMSTAR 2 appraisal criteria. While it is true that the reviews were not as rigorous or comprehensive as might be ideal, the weighting of this criterion could potentially be disputed. The AMSTAR 2 appeared to be overly stringent when used to appraise reviews in palliative care, which tend to include heterogenous designs and outcomes often found when evaluating complex interventions. We also acknowledge that this meta-review was not pre-registered on PROSPERO, owing to its origins in a student project. Although we use PDQ Levels of Evidence^36^ to categorise studies based on their design, we are aware that such frameworks value empirical over normative paradigms and may underestimate the contributions of qualitative research designs.

### Methodological gaps

Systematic review registration is not commonplace in palliative care but is needed to reduce potential for bias by reducing the opportunity for conscious or unconscious selection or manipulation of data to shape a review so that it reaches a desired conclusion. Both systematic reviews and primary studies within reviews need to consider sources of bias in palliative care research and describe how this is accounted for. The components of PICO should also be explicit, and comparison groups made clear in future reviews.

Our meta-review findings echo the wider literature on digital health^44^ and palliative care^45^, which point to the need for more rigorous evaluations, cost-effectiveness analyses, implementation studies and patient-centred research. The lack of rigorous cost-effectiveness studies seen in the literature on DHI in palliative care reflects findings from previous meta-reviews^46,47^ and systematic reviews^48–50^ in digital health. There is a need for greater clarity on what is being compared in cost-effectiveness studies, and whether the DHI is offered in addition to, or as a replacement for, the standard approach^14,26,35^. Undertaking large, well-powered RCTs on DHIs is challenging, partly because technological developments may outpace the timescale for conventional clinical trials^51^, and also because, in practice, DHIs are implemented in complex systems as opposed to controlled settings^52^. Complexity informed paradigms that take account of dynamic interactions occurring in the setting in which the DHI is being evaluated are needed, and methods that pay greater attention to the factors that facilitate or hinder adoption, such as in-depth case studies, may be more realistic and fruitful in future evaluations of DHIs for palliative care^51,53^. Evidence appraisal tools that value such methods are also required. Interdisciplinary evaluation, combining economic, social and clinical research, has the potential to better understand the role of different settings, healthcare needs and patient preferences for ensuring the appropriate, safe, acceptable and sustainable use of DHIs in palliative care. Early user involvement (patients, caregivers and staff) will also be a key in the design, evaluation and implementation of DHIs in this setting^54^.

### Technology evidence gaps

Personal health monitoring devices, such as wrist-worn activity trackers and smartwatches are now widely used and have been evaluated in other digital health contexts^55^. The absence of evidence about the use of these may reflect the fact that most studies of trackers are taking place in the context of chronic disease management. Nevertheless, it suggests a need for further research in palliative care, particularly for patients managing at home, for whom wearables and ambient computing (e.g. smart homes) are likely to be increasingly useful. The included systematic reviews did not include studies on the use of smartphone apps. Descriptive reviews on the potential that such apps may have in palliative care are emerging and further research is warranted^39,40^. Studies using machine learning and AI for risk detection and prediction, or for delivering personalised support based on the data from individual patients, were also not represented amongst the included reviews, despite progress in AI-enabled healthcare delivery^56^. Research exploring the use of machine learning using EHRs to predict mortality, and identify patients who would benefit from palliative care shows promise; future reviews need to consider this emerging evidence^57^. Studies involving robots or chatbots were not identified despite their potential application in palliative care^58^. Evidence on these types of DHIs is needed to understand their benefits and risks.

### Stakeholder evidence gaps

The WHO has developed a classification framework for DHIs, which provides a shared vocabulary for all stakeholders, including researchers, when evaluating effectiveness and identifying gaps in the implementation of DHIs across healthcare settings^59^. The WHO organizes DHIs into overarching categories by user group: clients (e.g. patients or caregivers), healthcare providers, health system or resource managers and data services. Most of the research evidence on DHIs in palliative care identified in this meta-review was focused on DHIs for healthcare providers (e.g. healthcare provider decision support, remote consultations; healthcare provider communication and training) and to a lesser extent for clients/patients (e.g. client-to-client communication via online peer group support). No research on interventions for health system managers or administrators in palliative care was found. Using the WHO framework to situate research on DHIs in palliative care, and identify gaps, facilitates engagement with the wider health and social care sector, and highlights the type of DHIs that may need to be prioritised for development and evaluation.

### Telemedicine and related evidence gaps

Most of the evidence identified in this meta-review focused on telemedicine, specifically remote consultations via phone and video. This evidence is timely as the Covid-19 pandemic has pivoted attention towards these approaches^60^. Remote consultations are feasible in palliative care and generally acceptable to patients^14,16,24,35^ and caregivers^18,35^. Remote consultations are perceived as particularly helpful when increasing access to care for families who are otherwise isolated by geography or housebound^14^, reflecting the context for many patients and families due to social distancing requirements during the Covid-19 pandemic. This should help reassure healthcare professionals that patients and caregivers often welcome these approaches, especially when face-to-face options are limited. While guidance regarding undertaking a remote consultation in palliative care is emerging^61^, evidence gaps remain. There is a need for research to determine when a face-to-face consultation is essential for terminally ill patients and when remote consultation is sufficient or preferred. Research is needed to understand contextual factors influencing the acceptability or effectiveness of remote consultations in palliative care^47^ and to shed light on inconsistent findings around physical and psychological symptoms found in the present review and in related research literature^62^. Critically, research on equitable access to palliative care delivered using DHIs is urgently needed to ensure that all those who need palliative care can benefit from it.

### Palliative care research participation

Research involving people who are terminally ill is difficult due to the perceived vulnerability of the population and professional caution^37^. Professional gatekeeping is a challenge^63^, and biased samples consisting of patients who are mostly well or highly motivated is often problematic. However, there is ample evidence that many terminally ill patients are interested in taking part in research and may benefit from doing so^64,65^. As patients and caregivers grow accustomed to receiving care remotely, there will be more opportunities to engage patients and their families in research remotely, reducing burden and travel costs. Providing a variety of ways in which patient and caregiver data can be collected, including online interviews and focus groups, maximises research participation, and is recommended.

## Conclusions

DHIs are increasingly being implemented in the context of palliative care and the Covid-19 crisis has given this further impetus, particularly for clinical and supportive interventions at a distance. This meta-review has synthesised the corpus of research evidence represented by existing systematic reviews in this area. The overall evidence suggests that DHIs can be useful, safe and acceptable to many terminally ill patients, their caregivers and staff involved in their care. Mostly, positive impacts were reported on education, information sharing, decision-making, communication and costs. Impacts on QoL and physical and psychological symptoms were inconclusive. Applying AMSTAR 2 criteria, most reviews were judged as low quality as they lacked a protocol or did not consider risk of bias, so findings need to be interpreted with caution.

Future meta-reviews would benefit from looser inclusion criteria to capture other types of reviews containing evidence on emerging innovations such as wearables, smartphone apps, robotics and artificial intelligence. Since the Covid-19 pandemic has greatly accelerated the use of novel digital health innovations and presents particular risks and barriers for the elderly and vulnerable, a large increase in published studies on new forms of service delivery may be expected in the coming year, which will call for new reviews of relevant evidence in palliative care.

## Methods

### Design

We undertook a systematic review of published systematic reviews, or a ‘meta-review’, to provide a single synthesis of relevant evidence on the use and effectiveness of DHIs for terminally ill patients and their families. Meta-reviews are useful in areas where numerous systematic reviews exist but vary in timeliness, scope and quality, making them difficult to interpret and use for evidence-based decision-making. Undertaking a meta-review typically involves applying a structured search strategy, filtering and critically appraising relevant systematic reviews, descriptive summarisation of the evidence base and thematic synthesis of the reviews’ findings and conclusions. Additional rigour can be provided by also critically appraising the primary studies contained within each review, although this is less common^66^. Our review considered systematic reviews as the main units of analysis. We nonetheless checked details of their included articles to identify the total number of unique publications, then classified these based on the type and purpose of the DHI reported, and the level of evidence suggested by their study designs.

### Protocol

A review protocol was developed in advance of conducting this meta-review and shared with the Research Ethics Subgroup in the Centre of Population Health Sciences at the University of Edinburgh. As this review started out as a postgraduate student project, it was not pre-registered on PROSPERO.

### Search strategy

The search strategy included the following databases: MEDLINE, MEDLINE In-Process & Other Non-Indexed Citations; EMBASE, PsychINFO, CINAHL, Cochrane Database for Systematic Reviews; Cochrane Database of Abstracts of Reviews of Effects; WHO Global Library (regional indexes only) and Web of Science. The Grey Literature Report (www.greylit.org) was also searched using keywords tailored for this database. The search terms included MeSH headings and keywords related to digital health, palliative care and technology. All search strategies can be found in the supplementary information file (see Supplementary Note 1). Searches were limited to articles published after 2006 to ensure relevance, given rapidly evolving technologies. There were no restrictions placed on language. The initial searches were conducted in June 2018; and subsequently extended, to capture additional studies published up to January 2020.

### Inclusion criteria

The search strategy targeted systematic reviews explicitly focused on DHIs in palliative care. Drawing on previous definitions of a systematic review, we included reviews with a clearly formulated question that uses systematic and explicit methods to identify, select and critically appraise relevant research^67^. We excluded rapid reviews and non-systematic scoping reviews, as well as reviews where search strategies were limited to one database, formal evidence appraisal was not undertaken, or data from individual studies were not summarized. Systematic reviews on broader topics were also included, provided these separately reported or synthesized studies of DHIs in palliative care. Using the PICO process^68^, we defined our target population (P) as children and adults who would benefit from palliative care, caregivers (informal and formal) and healthcare professionals delivering palliative care via DHIs or using DHIs to support palliative care decision-making. For the purposes of this review DHIs (I), were defined as approaches in which digital ICTs are used to deliver, facilitate or augment palliative care services, including psychological therapies, social support interventions, education, information, anticipatory care planning, remote care support, self-medication/management support, CDS etc. Examples of relevant ICTs include telephone, smartphone apps, mobile phones/SMS, videoconferencing, voice over IP, instant messaging, email, internet resources, tablets, wearables, electronic patient records. Both synchronous (e.g. videoconferencing) and asynchronous (e.g. email) approaches were included. Our comparator of interest (C) was no DHIs or usual care. No limitations were placed on outcomes (O), as we were interested in identifying the broad range of outcomes potentially influenced by palliative care DHIs.

### Data extraction

The co-first authors (A.M.F. and H.O’D.) undertook the database searches and initial screening of titles and abstracts. Where uncertainty existed in relation to potential eligibility, titles and abstracts were independently screened by a third author and ambiguities or disagreements resolved through discussion with the wider team. H.O’D. and A.M.F. independently assessed papers identified for full-text review, with CP arbitrating where it was unclear whether a review paper should be included. Disagreements and uncertainties were resolved during full team discussions and the authors came to a 100% agreement.

Three co-authors extracted the following information from each of the included systematic reviews: authors, date of publication, country, review aims, search strategy, number of studies included, total number of participants, definition of palliative care, details of participants, functions and medium of DHIs included, reported outcomes, quality assessment methods and conclusions. They then extracted the types of digital health technologies and the intended purposes of the technologies from the individual studies from the included reviews and sought advice from to a fourth co-author in cases of uncertainty.

We applied the PDQ Levels of Evidence framework described by the National Cancer Institute^36^ to appraise Supportive and Palliative Care research, to categorise publications within the systematic reviews into one of four levels. Level 1 represented study designs typically considered to offer the strongest evidence and Level 4 the weakest.

### Quality appraisal

The Assessment of Multiple Systematic Reviews (AMSTAR 2) checklist was used to critically appraise the included reviews^69^. This process was undertaken by three co-authors (A.M.F., J.L. and T.G.-W.). In advance of conducting the appraisal, each read the AMSTAR 2 guidance, and met to discuss the interpretation of tool for the present study. Two co-authors independently judged each review based on each of the 16 items identified in the AMSTAR 2. Three co-authors then met to compare item judgements for each of the 21 reviews. Final judgements were agreed through further discussion and consensus. Drawing on item judgements, an overall quality rating of the review was made based on the inclusion of AMSTAR 2 critical domains in each review. These judgements were based on the guidance described by Shea et al.^69^, which emphasizes an overall judgement of confidence based on critical and non-critical elements of the reviews, as opposed to calculating overall review scores. Given the heterogeneity of study designs in the included reviews, we viewed four domains as critical: protocol registered prior to commencement of the study (AMSTAR item 2), adequacy of the literature search (AMSTAR item 4), consideration of risk of bias when interpreting results (Amstar item 13) and discussion of heterogeneity observed in the results where relevant (Amstar Item 14). We rated quality based on overall confidence in the results of the review as follows: high: where all critical domains and no more than one non-critical weakness was evident, moderate: where all critical domains with few non-critical weaknesses were identified, low: where between one and two critical domains were not examined and very low: where three or more critical domains were not examined and numerous non-critical weaknesses were identified.

### Data synthesis

Based on our preliminary scoping work, we expected substantial heterogeneity amongst included reviews, several of which would themselves include a heterogeneous group of study designs. We therefore planned to categorise the key outcomes identified across all reviews during data extraction and undertake a narrative synthesis of the main findings related to these outcomes. All reviews were imported into NVivo 12, which we used to support the data analysis and synthesis process.

## Supplementary information

Supplementary Information

Supplementary Data 1

## Data Availability

Source data for all figure(s) and number(s) are provided with the paper and supplementary files. Aggregate data are available from the corresponding author on reasonable request.
