# Supplementary material for: Digital health interventions in palliative care: a systematic meta-review
Source: NPJ Digit Med. 2021 Apr 6;4:64. doi: 10.1038/s41746-021-00430-7 (PMC8024379; doi:10.1038/s41746-021-00430-7)
Supplement: Supplementary file 1 — Supplementary Information [file 41746_2021_430_MOESM1_ESM.pdf]

Supplementary materials

Supplementary Table 1:
Characteristics of the included systematic reviews Page 2

Supplementary Table 2:
Overall quality and findings from the included systematic reviews Page 11

Supplementary Table 3:
Assessment of Multiple Systematic Review (AMSTAR 2) ratings Page 28

Supplementary Note 1:
Database Search Strategies Page 31

Supplementary Table 1: Characteristics of the included systematic reviews (N=21)

Review	Country of institution	Broad area	Review aims and population	No. of included PC studies and types of studies	Type of DHIs included
**Willis et al. (2007)[27]**	USA	Internet	To examine internet use by hospice patients, their families, and hospice professionals.	**6 studies included**<br>* case study (n=1)<br>*questionnaire (n=1)<br>* internet surveys (n=2)<br>* descriptive narrative (n=1)<br>* review of case studies & survey (n=1)	* videophone<br>* digital pens<br>* online survey<br>* online chat room
**Kidd et al. (2010)[16]**	UK	Telehealth	To determine who is using telehealth and to what ends, and if the use of telehealth is increasing in the clinical setting.	**21 studies included**<br>* descriptive (n=9)<br>* pilot (n=3)<br>* service evaluation (n=1)<br>* system report (n=1)<br>* web resource (n=1)<br>* mixed methods (n=1)<br>*prospective cohort (n=1)<br>* qualitative (n=1)<br>* case study (n=1)<br>* not given (n=2)	* videoconferencing<br>* hand-held personal computer<br>*mobile phone<br>* website<br>* electronic records<br>* e-learning resource<br>*out-of-hours telephone advice and support services<br>* computer-based screening system<br>* computerised system<br>* NHS intranet

**Parker Oliver et al. (2012)[17]**	USA	Telehospice	To examine the evidence concerning tele-hospice services.	**26 studies included**<br>* surveys (n=6)<br>* mixed methods (n=10)<br>* interviews (n=4)<br>* not given (n=1)<br>* focus group (n=1)<br>* cost analysis (n=1)<br>* record review (n=1)<br>* content analysis (videotapes) (n=1)<br>* observation (n=1)	* telephone advice line<br>* videophones<br>* tele-hospice (technology not specified)<br>* PDAs<br>* computers & internet
**Zhou et al. (2012)[24]**	Canada	Telephone	To examine the use of telephone follow-ups compared with clinical follow-ups in advanced cancer patients receiving palliative care	**11 studies included**<br>* individual study designs not provided	* telephone follow-up consultations
**Bradford et al. (2013)[14]**	Australia	Telehealth	To examine the research of home-based tele-Health in paediatric PC.	**33 studies included**<br>* reviews (n=4)<br>* RCTs (n=4)<br>* cohort studies (n=2)<br>* chart reviews (n=2)<br>* cost comparison (n=1)<br>* quantitative survey (n=1)<br>* quantitative (n=1)<br>* mixed methods (n=2)<br>* qualitative (n=15)<br>* cost benefit analysis / qualitative (n=1)	* videoconferencing<br>* videophones<br>* SMS text message<br>* tele-Health<br>* telemedicine<br>* tele-hospice[15]

**Capurro et al. (2014)[15]**	Chile	Telehealth - asynchronous devices only	To identify eHealth interventions in PC and to determine the information requirements for patients, family members, and professionals in PC.	**17 studies included**<br>* quasi-experimental (n=3)<br>* observational; cross-sectional (n=11)<br>* qualitative inquiry (n=2)<br>* report (n=1)	* internet chat rooms<br>* mobile phones<br>* reporting software<br>* digital pens<br>* surveys<br>* telephone support<br>* PDAs
**Ngwenya and Mills (2014)[31]**	UK	Weblogs	To review evidence for benefits and disadvantages of weblogs in PC.	**6 studies included**<br>* online survey (n=2)<br>* case report (n=2)<br>* descriptive experimental study (n=1)<br>* analysis of blog posts (n=1)	* internet blogs<br>* online survey
**Allsop et al. (2015)[25]**	UK	ICT systems in general	To review existing Information and Communication Technology (ICT) systems created for pain management in oncology patients in PC.	**24 studies included**<br>* randomised experiment (n=3)<br>* non-randomised experiment (n=1)<br>* survey design (n=2)<br>* observational (n=1)<br>* expert opinion (n=1)<br>*non-experimental (n=13)<br>*description of system (n=2)<br>*Not described (n=1)	* tablet computer<br>* web-application software<br>* computer<br>* telephone<br>* handheld device<br>* mobile phone<br>* internet<br>* tablet<br>* PDA<br>* interactive touch screen<br>* online surveys<br>* pen tablet<br>* computer software<br>* voice-recorded telephone calls<br>* digital pen & pain diary<br>* e-tablet

**Chi and Demiris (2015)**[21]	USA	Telehealth	To review the effects of telehealth applications on caregivers. (A subset of studies were PC focused)	**5 studies included**<br>* quasi-experimental (n=2)<br>* comparison case study (n=1)<br>* pilot-controlled trial without randomisation (n=1)<br>* 1 RCT	* videophones<br>* home telehealth service
**Ostherr et al. (2016)**[26]	USA	ICT systems in general	To review the use of ICTs in end-of-life care for communication between clinicians, patients and their families (any setting).	**38 studies included**<br>* RCT (n=17)<br>* pre-post interventions (n=18)<br>* interrupted time series (n=2)<br>* prospective cohort study (n=1)	* videos<br>* online support system<br>* online education<br>* prototype websites<br>* videoconferencing<br>* computer-based multimedia decision tool<br>* CD<br>* telephones<br>* videotape<br>* SMS text messaging<br>* telemonitoring<br>* web-based PC report card<br>* fax<br>* palm pilot

**Zheng et al. (2016)[18]**	USA	Telehealth	To review telehealth interventions on caregivers in palliative care settings.	**9 studies included**<br>* prospective cohort (n=1)<br>* mixed methods (n=2)<br>* pooled analysis of 2 RCTs (n=1)<br>* non-randomized pre-post test (n=1)<br>* pilot study (n=1)<br>* mixed methods case study (n=1)<br>* randomised noninferiority study (n=1)<br>* feasibility study (n=1)	* home telehealth consultations<br>* videophones<br>* telehealth<br>* online support system<br>* telephones
**Head et al. (2017)[30]**	USA	Telehealth	To describe patient-reported outcomes in telehealth studies for palliative care	**11 studies included**<br>*RCT with pre-test and post-test design (n=1)<br>*quantitative survey (n=1)<br>*mixed methods (n=1)<br>*mixed methods with semi-structured interviews (n=1)<br>*qualitative case study (n=1)<br>*case study (n=1)<br>*mixed methods case study (n=1) *pre/post survey (n=1)<br>*case report (n=1)<br>randomized *non inferiority trial (n=1)<br>*two-group non-randomised study (n=1)	* telephone<br>* WhatsApp<br>* videophones<br>* text messaging<br>* mobile phone-based symptom assessment software<br>* home telehealth monitoring<br>* Rexnet – computer program to enable text messages between nurses and patients

**Taroco et al. (2017)[23]**	Brazil	Internet	To review online educational PC programs to update health professionals	**14 studies included**<br>Types of studies not provided.	* video conference<br>* internet
**Allsop et al. (2018)[33]**	UK	mHealth	To identify the use and development of mHealth in PC in sub-Saharan Africa.	**5 studies included**<br>*Rapid evaluation methodology (n=1)<br>*Interviews (n=1)<br>*Survey and follow-up interview (n=1)<br>*Pre-and post-test survey (n=1)<br>*Mixed method (n=1)	* mobile phones<br>* email<br>* telephone<br>* electronic health system<br>* instant messaging<br>* blog<br>* text messages
**Bush et al (2018)[28]**	USA	Electronic Health Records	To determine whether Electronic Health Records (EHRs) and related Clinical Decision Supports facilitate PC.	**30 studies included**<br>*Feasibility (n=9)<br>*Retrospective cohort analysis (n=5)<br>*Mixed methods (n=5)<br>*Secondary analysis (n=4)<br>*Surveys (n=2)<br>*Qualitative interviews (n=3)<br>*Retrospective analysis (n=1)<br>*Pilot (n=1)	Electronic Health Records
**Smith et al (2018)[22]**	USA	Simulators	To review the use of simulation-based learning experiences (SBLEs) to teach communication skills to nursing students and clinicians who provide	**30 studies included**<br>*Not described	* high fidelity and medium fidelity simulators<br>* video

			palliative and end-of-life care to patients and their families.		
**Huber et al (2018)[29]**	USA	Electronic Health Records	To review the literature for electronic health record (EHR) interventions to improve ACP documentation; To describe the EHR components of these interventions; to identify populations in which the interventions were implemented; to assess the efficacy of the interventions in these populations.	**16 Studies included**<br>*Comparative analyses (n=9)<br>*RCT (n=1)<br>*non-randomized trials (n=3)<br>*Pre–post analyses (n=3)	*electronic Health Records

**Hancock et al (2019)[33]**	UK	Telehealth	To describe the current use of telehealth in palliative care in the UK and evaluate telehealth initiatives against a digital service standard.<br><br>To explore whether telehealth results in a reduction in emergency care access	**30 papers relating to 27 studies.**<br>*Qualitative (n=7)<br>*Service evaluations (n=4)<br>*Randomized controlled trials (n=3)<br>*Protocols (n=3)<br>*Descriptive (n=3)<br>*Randomized crossover trial (n=1)<br>*Mixed methods (n=2)<br>*Realist evaluation (n=1)<br>*Prospective interventional (n=1)<br>*Prospective longitudinal cohort (n=1)<br>*2 Prospective observational (n=2)<br>*2 Retrospective observational (n=2)	*home telemonitoring<br>*videoconferencing<br>*telephone advice line<br>*electronic patient records<br>*tablets/apps
**Jess et al (2019)[35]**	Denmark	Video-consultations	To review evidence for video consultations in palliative care from the perspective of patients and relatives, health care professionals, and society.	**39 articles included.**<br>*mixed methods (n = 14)<br>*qualitative (n = 10)<br>*quantitative (n = 10) including one RCT<br>*case studies (n = 5).	*Video-consultations

**Leniz et al (2019)[10]**	UK	Electronic Health Records	To review evidence on electronic palliative care co-ordination systems (EPaCCS), in order to identify gaps in the evidence and make recommendations for policy and research.	**12 studies included**<br>*observational studies (n=9)<br>*qualitative studies (n=2)<br>*mixed-method (n=1).<br>(18 opinion pieces and reports excluded)	*Electronic palliative care coordination systems
**Lemon et al (2019)[34]**	Australia	Electronic Health Records	To identify and evaluate the evidence for using EMRs (electronic medical records) in documenting advanced directives (AD) and identifying implications of the findings for addressing challenges in their use.	**15 studies**<br>*4 Pre-post<br>*4 cross-sectional<br>*2 retrospective<br>*1 RCT<br>*1 RCT pilot<br>*1 historical control<br>*1 retrospective cohort<br>*1 Evaluation of EMR	Electronic Medical Records (EMRs)

Supplementary Table 2: Overall quality and findings from the included systematic reviews (N=21)

Review authors	AMSTAR 2	Critical appraisal tools used	Quality of included studies	Review findings	Summary
**Willis et al. (2007)** [27]	Very low	None	Not assessed.	* improved communication between patients, caregivers, and professionals<br>* improves continuity of care<br>* patients perceived improved quality of care<br>* reasons for health professionals to use internet: email, online journals, finding clinical information<br>* patients seek information online about their illness and alternative treatments<br>*patients like the anonymity of online support groups	Internet-based interventions are effective for patients and professionals who use the web to search for answers to medical questions, deliver interventions, and for communication purposes.
**Kidd et al. (2010)**[16]	Very low	None	Not assessed	* improved continuity of care<br>* improved clinical effectiveness<br>* reduced costs<br>* effective use of resources<br>* useful for education and disseminating practice guidelines<br>* acceptable to patients and health professionals	Patients and health professionals find DHIs acceptable and usable in palliative care. Preliminary evidence for effectiveness though barriers remain to integrating DHIs into routine practice.

				* feasible alternative when distance, time, and costs are restraints<br>* lack of evidence-based research	
**Parker Oliver et al. (2012)[17]**	Low	Two-part self-developed scoring framework	Mean quality score for quantitative studies was 9.2 (range 5-14), representing medium-high strength of evidence.<br><br>Mean quality score for qualitative studies was 9 out of 11, representing medium to high strength of evidence.	* hospice providers supportive of tele-hospice technologies<br>* no study was large enough to demonstrate significant differences in patient anxiety, caregiver QOL, communication, anxiety, and caregiver thoughts on pain medication<br>* majority of studies were small, reflecting fledgling nature of research area<br>* barriers to implementation: included gatekeeping and variation in staff member's readiness and ability to use DHIs.	Studies have evaluated the use of a variety of technologies, attitudes toward use by providers and consumers, clinical outcomes, barriers, readiness, and cost.<br>The evidence base, although growing, is of mixed scientific rigor with lower-medium strength evidence in quantitative studies and medium-higher strength evidence in qualitative studies.<br>Barriers to implementation were evident.
**Zhou et al. (2012)[24]**	Low	None	Not assessed	* feasible to take clinical information over the phone<br>* minimised burden on patient to attend clinics *maintained	Telephone follow-ups provide an acceptable and feasible alternative to in-person clinical follow-ups for assessing

				QOL<br>* poor accrual in clinical studies noted as a problem in advanced cancer populations<br>*attrition rates still a problem with telephone follow-ups<br>* reduces burden on care facility<br>* improves contact with poor performance status patients<br>* positive opinion of intervention in general and no disadvantages were noted<br>* combining in-person clinical follow ups and telephone follow-ups could provide a more complete assessment	patient symptoms, decreasing burden, and enabling quality of life to be maintained.
**Bradford et al. (2013)[14]**	Low	Critical Appraisal Skills Programme (CASP)	Moderate to high.<br><br>CASP scores ranging from 4/8 to 11/11 depending on study design.	* Home telehealth generally acceptable for families and clinicians as part of the palliative care provided<br>* feasible for delivering care<br>* effects on QOL and anxiety positive overall<br>* decrease in parental anxiety<br>* increased confidence levels of families<br>* some evidence for cost-effectiveness<br>* no adverse effects<br>* improves access to care	Studies generally identified benefits of using home telehealth in palliative care and overall evidence was judged moderate to high quality. However, research on DHIs in paediatric palliative care is challenging. More research is needed to assess what influences acceptance of these DHIs, including ease of utilizing the technology and care goals

				* reduces travel costs for patients and caregivers<br>* issues: impersonal, no human contact, financial issues regarding reimbursement<br>* economic limit to eHealth interventions if intervention supplements rather than replaces the standard care<br>* barriers to use: clinicians are gatekeepers and healthcare settings need to be ready for eHealth interventions<br>*implementation: must fully engage with all staff and patients, patient centred approach, and clinician to champion eHealth<br>* Several methodological challenges in conducting research in paediatric palliative care.	

**Capurro et al. (2014)[15]**	Very low	None	Not assessed	* telephone advice lines provided support with pain management, managing symptoms, and medication usages<br>* digital pens improved communication with caregivers and quality of care<br>* some evidence that DHIs decreased hospitalisations, emergency care visits, bed days, and reduced costs for veteran patients & their families<br>* mobile phone-based technology useful for detecting symptoms earlier<br>*Robust clinical trials needed.	There was heterogeneity in the types of interventions and outcomes assessed. Some studies reported some improvement on quality of care, documentation effort, cost, and communication. Overall inadequate evidence on effectiveness.
**Ngwenya and Mills (2014)[31]**	Very low	None	Not assessed	* blogging can be therapeutic and enable people to self-reflect<br>* enables people to openly express feeling and opinions<br>* creates sense of identity and connection online<br>* theme of empowerment identified<br>* communication: new way for patients and hospice staff to communicate<br>* blogs are a low-cost intervention	Weblogs were found to be helpful and therapeutic to PC bloggers, providing social support which may help improve well-being. There was a lack of rigorous evidence demonstrating the advantages of weblogs in palliative care. More research is needed to assess the benefits and effectiveness. Given the small number of small-scale studies, overall strength of evidence is likely to be low.

**Allsop et al. (2015)** [25]	Low	None	Not assessed	* majority of studies were non-specified non-randomised<br>* no consistent measurement tools used across studies.<br>* ICT helps with gathering clinical information before consultation<br>* provides flexibility of symptom reporting<br>* 2 types of communication identified: patient to health professional with no feedback and patient to health professional with feedback after health professional reviews information.<br>* communication: systems for facilitating communication did not increase communication between patient and health professionals	ICT systems for symptom reporting<br>are emerging in the palliative care context. Most are at an early stage of development. There is a need to increase the quality and scale of development work and explore how to effectively use system feedback with patients.
**Chi and Demiris (2015)**[21]	Very low	Oxford Centre for Evidence-based Medicine framework	Medium to high quality	* improved communication<br>* improved satisfaction levels<br>* improved anxiety levels and QOL<br>* improved problem-solving abilities<br>* 1 study showed no differences in parental QOL between control group and telehealth group	Telehealth provides positive effects for caregivers of people with chronic diseases who are receiving palliative care, including improved satisfaction, quality of life and psychological wellbeing. Findings are based on a sub-sample of studies reported in the full paper.

**Ostherr et al. (2016)[26]**	Low	Cochrane risk of bias tool	Studies were judged to be at medium to high risk of bias, mainly due to non-blinding of participants and outcomes, and small sample sizes.	* ICTs were most commonly used to provide information or education,<br>serve as decision aids, promote advance care planning (ACP), and relieve physical symptom distress<br>*Over half of all included studies used video, and the evidence base for the use of video in EOL communication was judged as strong.<br>*Video was an effective decision support tool for ACP.<br>*Video were useful for education and communication purposes.	Evidence for the use of video in end-of-life care communication was judged as strong, though overall DHI evidence was judged to be at medium to high risk of bias. Several studies demonstrated the efficacy of video as a decision support tool in ACP. Few studies involving mobile and connected platforms. Further research based on mobile technologies is needed.

**Zheng et al. (2016)[18]**	Low	Cochrane risk of bias tool	Of the nine studies, the majority (77.8%) were judged as moderate quality. Only two of the nine studies reported a randomized process of participant recruitment and allocation, and none reported using a process for blinding participants.	* 5 studies on QOL did not show any significant differences between the intervention and control groups<br>* 4 studies reported telehealth interventions are feasible<br>* 5 studies found caregivers' to be satisfied with the telehealth intervention<br>* Decreased physical QOL in one study<br>* 2 studies show significant decrease in anxiety levels after the intervention, but 1 showed no significant improvement<br>* 1 study reported reduced burden on caregivers<br>* 1 study did not show any significant differences on burden levels<br>* 1 study found improved family functioning<br>* 1 study found online symptom reporting reduced negative mood<br>* 1 study found decreased depression and perceived stress over time	Some evidence of overall satisfaction in caregivers who use a telehealth intervention, but outcomes reported were often not substantial. Overall study quality was judged moderate, but methodological flaws and small sample sizes negatively were problematic. More rigorous research is needed.

**Head et al. (2017)**[30]	Low	Cochrane risk of bias tool	Of the 6 studies reporting quantitative outcomes, 3 studies were moderate quality and 3 were low quality.<br><br>Of the six qualitative studies, quality scores ranged from 2 to 5 out of a possible score of 11. Information was lacking on several dimensions which may have contributed to the low scores.	* 4 studies reported positive patient satisfaction with the eHealth intervention<br>* 2 studies measured QOL, 1 showed no significant difference while other showed positive effects<br>* 3 studies reported improvement symptoms after intervention, 1 found no effect.<br>* 2 studies reported decreased anxiety and depression<br>*2 studies found decreased hospital costs due to eHealth intervention	All studies, except one, reported positive results for eHealth interventions. But overall evidence for positive patient outcomes in palliative telehealth interventions was weak. There was wide variability across the studies in terms of patient population, outcomes measured, methodology, and technology used. Lack of standardised outcomes alongside recruitment challenges and attrition made evaluation difficult.

**Taroco et al. (2017)[23]**	Low	None	Not assessed	* majority of educational initiatives aimed at nurses and HPs<br>* half of overall courses focused on PC as a general topic<br>* courses lasted between 2-6 months on average<br>* most courses were mixed in their delivery approach<br>* half of the courses used pretest and post-tests and the other half used only post-tests<br>* online mixed teaching methods enable practical and theoretical activities in a cost-effective manner which helps healthcare settings with limited resources<br>*Evaluation of distance learning outcomes was not undertaken.	Evaluation outcomes were not reported. Limited research exploring the construction process of courses and how they can be applied to countries with limited resources was identified.

**Allsop et al. (2018)**[33]	Low	None	Not assessed	* Health workers in remote areas were finding innovative ways to use mHealth technologies.<br>* mHealth technologies used to improve appointment adherence in resource limited areas and facilitate communication with patients and families<br>* mHealth used for information and educational purposes.<br>*Short term lectures and informational text messages can help empower physicians<br>*Patients preference mobile phone over travelling long distances	Existing mHealth interventions in sub-Saharan Africa were limited in number and used at the palliative treatment, guidance, and coordination stage of care provision. There was a lack of information relating to mHealth infrastructure requirements, technology and platforms used, and costs related to delivering the intervention was noted.
**Bush et al (2018)**[28]	Low	None	Not assessed	5 major areas in which the EHR is used to support palliative care were identified:<br>*identifying individuals who could benefit from palliative care<br>*enhancement of the EHR to improve palliative care<br>* advance care planning (ACP) documentation;<br>*patient-reported outcomes such as rapid, real-time pain feedback	Studies focused on clinical decision systems to: identify individuals who could benefit from PC; facilitate electronic advanced care planning (ACP) documentation; improve patient-reported outcome measures (PROMs); to augment EHR PC data capture capabilities; and to enhance interdisciplinary communication and care. The use of EHRs and clinical

				*enhancing interdisciplinary communication.	decision system are underutilised despite some evidence for their usefulness.
**Smith et al (2018)[22]**	Low	Kirkpatrick's level of evaluation was used to differentiate the level of evaluation assessment completed.	Lack of standardization, poor evaluation methods, and limited exposure to the entire interprofessional team made it difficult to identify and disseminate validated best practices.	*Simulation-based learning is being used to teach palliative and end of life care.<br>*High fidelity simulators are the most common technology used.<br>*Most simulation-based learning experiences are supported by video review.<br>* The wide variety and heterogeneity of simulation-based learning experiences made it difficult to draw conclusion on effectiveness.	Simulation-based learning experience (SBLE) are being used to teach palliative and end-of-life communication skills to nursing students and clinicians. Lack of standardization, poor evaluation methods, and limited exposure to the entire interprofessional team makes it difficult to identify and disseminate validated best practices. Further research is needed employing rigorous evaluation methods and measures that link the SBLE to the training objectives and desired clinician practice

					behaviours and patient outcomes. Quality assessment of primary studies was not undertaken.
**Huber et al (2018)[29]**	Low	None	Not assessed	*The most common EHR interventions described were documentation templates, followed by prompts and electronic order sets.<br>*Documentation templates can reduce variability in documentation and gather information associated with high quality ACP<br>*All studies reporting efficacy (n=7) reported an improvement in one or more ACP outcomes.	EHR interventions, such as documentation templates, order sets, and prompts, may improve the incidence and quality of ACP and may improve ACP completion and availability at the point of care.

**Hancock et al (2019)**[33]	Low	Critical appraisal based on criteria adapted from Wallace (2004).	Eight of the 19 papers met all the nine criteria completely or to some extent.<br>11 papers did not meet the nine criteria because of insufficient sample sizes or insufficient description of data collection methods.	*Telehealth was used to support patients and carers, monitor symptoms and provide education.<br>*The number of home telemonitoring initiatives for patients had increased since 2010.<br>*Many studies were small scale, descriptive and provided little evidence of evaluation of the service.<br>*Seven included studies made specific reference to reduction in access to emergency or acute care services.	Telehealth was used to support patients and carers, electronic record keeping and professional education. However, many studies were small scale, descriptive and provided little evidence of evaluation of the service. There remains a lack of evaluation and robust study design, meaning conclusions regarding the clinical application of telehealth in palliative care cannot be drawn. There is insufficient evidence to appreciate any benefit of telehealth on access to emergency care.

**Jess et al (2019)[35]**	Moderate	Quality assessment was based on a tool developed by Hawker et al. (2002). Each study could receive between 10 and 40 points.	Studies scored between 20 and 36 points with an average score of 27.4 points (possible range 10-40).<br><br>Studies scored lowest in ethics and bias, as these criteria were often inadequately reported.	*Video-consultations enabled patients and/or relatives to connect with one or more health professionals in different locations enabling wider participation, reductions in travel burden, and a reduction in emergency admissions.<br>*Video consultations enabled communication as several participants could be visually present and communicate with each other and resolve issues in one consultation. Enabled shared decision-making and facilitated carer involvement.<br>* Most patients, relatives and health care professionals were positive towards the technology which provided a feeling of security.<br>*User friendliness was a facilitator.<br>*Technical challenges were a barrier to uptake.<br>*Some patients were reluctant to give health care professionals visual access to their home.<br>*3 out of 4 studies found that video consultations had	Video consultations enable clinical assessment and support and effective verbal and non-verbal communication at a distance. However, evidence beyond specialized palliative care and patients with cancer is limited. Some studies report cost-savings and most users were positive towards the technology. Technical challenges could be a barrier and there are implications for patients' privacy and security. Future research should focus on how and when video consultations might replace in-person specialized palliative care and video consultations in general palliative care, in low- and middle-income countries; and involving patients with a non-cancer diagnosis.

				economic advantages for providers or patients.	
**Leniz et al (2019)[10]**	Low	Standard Quality Assessment Criteria (Kmet et al 2004) for evaluation of primary research papers from different fields.	Mean quality appraisal score for quantitative studies was 85%. The most common source of poor quality for quantitative studies was the lack of an evident and appropriate study design and a poorly defined comparison group.<br><br>Mean quality	*Place of death: 4 studies reported the proportion of people who died in their preferred place with a shared electronic record (55% to 79%) was higher than the average for the population.<br>*Two of the highest quality studies found that EPaCCS use was associated with lower odds of hospital death, hospital admission and emergency department attendance<br>*Qualitative evidence found that EPaCCS are generally acceptable for patients and healthcare professionals. However, in-hours staff	Much of the current scientific literature on EPaCCS comprises of expert opinion. There is an absence of experimental studies evaluating the impact of EPaCCS on end-of-life outcomes. Further rigorous evaluations of EPaCCS, including economic impacts are needed.

			appraisal score for qualitative studies was 83%. The most common source of poor quality for qualitative studies was a lack of reflexivity of the account and description of the theoretical framework.	perceived EPaCCS as a potential burden due to an increased workload without perceivable benefit to them, while out-of-hours staff perceived EPaCCS to be more useful.<br>*Only two studies sought patient perspectives.	
**Lemon et al (2019)[34]**	Low	Cochrane risk of bias tool	Most studies had an unclear or high risk of bias.	*7 studies showed that EMR-based reminders, advance directive (AD) templates, and decision aids can improve AD documentation rates.<br>*3 demonstrated that EMR search functions, decision aids, and automatic identification software can help identify patients who have or need ADs.<br>*5 showed EMRs can create documentation challenges, including locating ADs, and making some patients more likely than others to have an AD.	Limited evidence suggests electronic medical records could be used to help address advance directive documentation challenges but may also create additional problems. Stronger evidence is needed to determine how electronic medical records more conclusively may assist in population approaches to improving advance directive documentation.

Supplementary Table 3

AMSTAR 2 Item	AMSTAR 2 Item Description*	Willis et al. (2007)	Kidd et al. (2010)	Parker Oliver et al. (2012)	Zhou et al. (2012)	Bradford et al. (2013)	Capurro et al. (2014)	Ngwenya and Mills (2014)
1	Did the research questions and inclusion criteria for the review include the components of PICO?	No	No	No	No	No	No	No
2	Did the report of the review contain an explicit statement that the review methods were established prior to the conduct of the review and did the report justify any significant deviations from the protocol?	No	No	No	No	No	No	No
3	Did the review authors explain their selection of the study designs for inclusion in the review?	No	No	Yes	Yes	Yes	Yes	Yes
4	Did the review authors use a comprehensive literature search strategy?	Partial yes	Partial yes	Partial yes	Partial yes	Partial yes	Partial yes	Partial yes
5	Did the review authors perform study selection in duplicate?	Partial yes	Yes	No	Yes	No	Yes	Yes
6	Did the review authors perform data extraction in duplicate?	No	Yes	No	Yes	No	Yes	Yes
7	Did the review authors provide a list of excluded studies and justify the exclusions?	No	No	No	No	No	Yes	No
8	Did the review authors describe the included studies in adequate detail?	No	Partial yes	Yes	Yes	Yes	Partial yes	Partial yes
9	Did the review authors use a satisfactory technique for assessing the risk of bias (RoB) in individual studies that were included in the review?	No	No	No	No	Yes	No	No
10	Did the review authors report on the sources of funding for the studies included in the review?	No	No	Yes	No	No	No	No
11	If meta-analysis was performed did the review authors use appropriate methods for statistical combination of results?	No meta analysis conducted	No meta analysis conducted	No meta analysis conducted	No meta analysis conducted	No meta analysis conducted	No meta analysis conducted	No meta analysis conducted
12	If meta-analysis was performed, did the review authors assess the potential impact of RoB in individual studies on the results of the meta-analysis or other evidence synthesis?	No meta analysis conducted	No meta analysis conducted	No meta analysis conducted	No meta analysis conducted	No meta analysis conducted	No meta analysis conducted	No meta analysis conducted
13	Did the review authors account for RoB in individual studies when interpreting/ discussing the results of the review?	No	No	Yes	No	Yes	No	No
14	Did the review authors provide a satisfactory explanation for, and discussion of, any heterogeneity observed in the results of the review?	Yes	No	Yes	Yes	Yes	No	No
15	If they performed quantitative synthesis did the review authors carry out an adequate investigation of publication bias (small study bias) and discuss its likely impact on the results of the review?	No meta analysis conducted	No meta analysis conducted	No meta analysis conducted	No meta analysis conducted	No meta analysis conducted	No meta analysis conducted	No meta analysis conducted
16	Did the review authors report any potential sources of conflict of interest, including any funding they received for conducting the review?	Funding only	Funding only	Funding/CoI	Funding/CoI	Funding/CoI	Funding/CoI	Funding
	**Rating based on AMSTAR 2 criteria**	**Very low**	**Very low**	**Low**	**Low**	**Low**	**Very low**	**Very low**

AMSTAR 2 Item	AMSTAR 2 Item Description*	Allsop et al. (2015)	Chi and Demiris (2015)	Ostherr et al. (2016)	Zheng et al. (2016)	Head et al. (2017)	Taroco et al. (2017)	Allsop et al. (2018)
1	Did the research questions and inclusion criteria for the review include the components of PICO?	No	No	No	No	No	No	No
2	Did the report of the review contain an explicit statement that the review methods were established prior to the conduct of the review and did the report justify any significant deviations from the protocol?	No	No	No	No	No	No	No
3	Did the review authors explain their selection of the study designs for inclusion in the review?	Yes	Yes	Yes	Yes	Yes	Yes	Yes
4	Did the review authors use a comprehensive literature search strategy?	Partial yes	Partial yes	Partial yes	Partial yes	Partial yes	Partial yes	Partial yes
5	Did the review authors perform study selection in duplicate?	No	Yes	Yes	Yes	Yes	Yes	No
6	Did the review authors perform data extraction in duplicate?	No	No	Yes	Yes	Yes	Yes	No
7	Did the review authors provide a list of excluded studies and justify the exclusions?	No	No	No	No	No	No	No
8	Did the review authors describe the included studies in adequate detail?	Yes	Yes	Yes	Yes	Yes	Partial yes	Partial yes
9	Did the review authors use a satisfactory technique for assessing the risk of bias (RoB) in individual studies that were included in the review?	No	No	Yes	Yes	Yes	No	No
10	Did the review authors report on the sources of funding for the studies included in the review?	No	No	No	No	No	No	No
11	If meta-analysis was performed did the review authors use appropriate methods for statistical combination of results?	No meta analysis conducted	No meta analysis conducted	No meta analysis conducted	No meta analysis conducted	No meta analysis conducted	No meta analysis conducted	No meta analysis conducted
12	If meta-analysis was performed, did the review authors assess the potential impact of RoB in individual studies on the results of the meta-analysis or other evidence synthesis?	No meta analysis conducted	No meta analysis conducted	No meta analysis conducted	No meta analysis conducted	No meta analysis conducted	No meta analysis conducted	No meta analysis conducted
13	Did the review authors account for RoB in individual studies when interpreting/ discussing the results of the review?	No	No	Yes	Yes	Yes	No	No
14	Did the review authors provide a satisfactory explanation for, and discussion of, any heterogeneity observed in the results of the review?	Yes	No	Yes	Yes	Yes	Yes	Yes
15	If they performed quantitative synthesis did the review authors carry out an adequate investigation of publication bias (small study bias) and discuss its likely impact on the results of the review?	No meta analysis conducted	No meta analysis conducted	No meta analysis conducted	No meta analysis conducted	No meta analysis conducted	No meta analysis conducted	No meta analysis conducted
16	Did the review authors report any potential sources of conflict of interest, including any funding they received for conducting the review?	Funding/CoI	Funding	Funding/CoI	CoI	No	Funding/CoI	Funding/CoI
	**Rating based on AMSTAR 2 criteria**	**Low**	**Very low**	**Low**	**Low**	**Low**	**Low**	**Low**

AMSTAR 2 Item	AMSTAR 2 Item Description*	Bush et al (2018)	Smith et al (2018)	Huber et al (2018)	Hancock et al (2019)	Jess et al (2019)	Leniz et al (2019)	Lemon et al (2019)
1	Did the research questions and inclusion criteria for the review include the components of PICO?	No	No	Yes	Yes	No	No	No
2	Did the report of the review contain an explicit statement that the review methods were established prior to the conduct of the review and did the report justify any significant deviations from the protocol?	No	**Yes**	No	**Yes**	**Yes**	No	No
3	Did the review authors explain their selection of the study designs for inclusion in the review?	Yes	Yes	Yes	Yes	Yes	Yes	Yes
4	Did the review authors use a comprehensive literature search strategy?	Partial yes	Partial yes	Partial yes	Partial yes	Partial yes	Partial yes	Partial yes
5	Did the review authors perform study selection in duplicate?	Yes	Yes	Yes	Yes	No	Yes	Yes
6	Did the review authors perform data extraction in duplicate?	Yes	Yes	Yes	Yes	No	Yes	Yes
7	Did the review authors provide a list of excluded studies and justify the exclusions?	No	Partial yes	No	No	No	Yes	No
8	Did the review authors describe the included studies in adequate detail?	Partial yes	Partial yes	Partial yes	Partial yes	Yes	Yes	Partial yes
9	Did the review authors use a satisfactory technique for assessing the risk of bias (RoB) in individual studies that were included in the review?	No	No	No	No	Yes	Yes	Yes
10	Did the review authors report on the sources of funding for the studies included in the review?	No	No	No	No	No	No	No
11	If meta-analysis was performed did the review authors use appropriate methods for statistical combination of results?	No meta analysis conducted	No meta analysis conducted	No meta analysis conducted	No meta analysis conducted	No meta analysis conducted	No meta analysis conducted	No meta analysis conducted
12	If meta-analysis was performed, did the review authors assess the potential impact of RoB in individual studies on the results of the meta-analysis or other evidence synthesis?	No meta analysis conducted	No meta analysis conducted	No meta analysis conducted	No meta analysis conducted	No meta analysis conducted	No meta analysis conducted	No meta analysis conducted
13	Did the review authors account for RoB in individual studies when interpreting/ discussing the results of the review?	No	No	No	No	Yes	Yes	Yes
14	Did the review authors provide a satisfactory explanation for, and discussion of, any heterogeneity observed in the results of the review?	Yes	Yes	Yes	Yes	Yes	Yes	Yes
15	If they performed quantitative synthesis did the review authors carry out an adequate investigation of publication bias (small study bias) and discuss its likely impact on the results of the review?	No meta analysis conducted	No meta analysis conducted	No meta analysis conducted	No meta analysis conducted	No meta analysis conducted	No meta analysis conducted	No meta analysis conducted
16	Did the review authors report any potential sources of conflict of interest, including any funding they received for conducting the review?	Funding	Funding/CoI	Funding/CoI	Funding/CoI	Funding/CoI	Funding/CoI	Funding/CoI
	**Rating based on AMSTAR 2 criteria**	**Low**	**Low**	**Low**	**Low**	**Moderate**	**Low**	**Low**
	Note: Critical items are highlighted in yellow							

Supplementary Note 1

Database Search Strategies

EMBASE

1 palliat*.mp.
2 ("end-of-life" or "end of life").mp.
3 "last year of life".mp.
4 exp terminal care/
5 exp terminal disease/ or exp terminally ill patient/ or terminally ill.mp. or terminal illness.mp. or terminal disease.mp.
6 exp hospice/ or exp hospice care/ or exp hospice/ or exp hospice nursing/ or exp hospice patient/ or hospice.mp.
7 care home*.mp.
8 exp nursing home/ or nursing home*.mp.
9 supportive care.mp.
10 supportive oncology.mp.
11 ((advanced or "late-stage" or "end-stage") and (disease or illness or carcinoma or neoplasm) and ("symptom control" or pain or cachexia or breathlessness or distress)).mp.
12 ("end-stage" and (heart or liver or cardiac or kidney or renal)).mp.
13 (dementia and (advanced or "late-stage")).mp.
14 1 or 2 or 3 or 4 or 5 or 6 or 7 or 8 or 9 or 10 or 11 or 12 or 13
15 ehealth/ or e-health.mp. or e*health.mp.
16 exp telemedicine/ or telemedicine.mp.
17 exp telehealth/ or telehealth.mp.
18 mhealth/ or m-health.mp. or m*health.mp.
19 exp telephone/ or telephone*.mp.
20 exp mobile phone/ or mobile phone*.mp. or cell phone*.mp.
21 (smartphon* or iphone*).mp.
22 (text messag* or short messaging service or sms).mp.
23 exp electronic medical record/ or electronic health record*.mp. or electronic medical record*.mp.
24 patient health record*.mp.
25 personal health record*.mp.
26 electronic health.mp.
27 exp teleconsultation/ or teleconsult*.mp. or tele*consult*.mp.
28 telecare.mp.
29 exp medical informatics/
30 exp computer network/ or computer communication network.mp.
31 exp telecommunication/ or telecommunicat*.mp.

32 "information and communication technolog*".mp.
33 exp videoconferencing/ or videoconsult*.mp. or video consult*.mp. or videocommunicat*.mp. or video communicat*.mp. or skype.mp. or facetime.mp.
34 (laptop tablet* or tablet computer * or ipad*).mp.
35 exp telemonitoring/ or telemonitor*.mp.
36 ecommuncat*.mp.
37 exp teleconsultation/ or teleconsult*.mp.
38 15 or 16 or 17 or 18 or 19 or 20 or 21 or 22 or 23 or 24 or 25 or 26 or 27 or 28 or 29 or 30 or 31 or 32 or 33 or 34 or 35 or 36 or 37
39 review*.mp.
40 meta-analysis.mp.
41 exp "systematic review"/ or systematic* review*.mp. or systematic literature review*.mp.
42 exp evidence based medicine/
43 evidence synthesis.mp.
44 best practice*.mp.
45 systematic.m_titl.
46 39 or 40 or 41 or 42 or 43 or 44 or 45
47 14 and 38 and 46
48 limit 47 to yr="2006 -Current"

MEDLINE

1 palliat*.mp.
2 (end-of-life or end of life).mp.
3 last year of life.mp.
4 exp Terminal Care/
5 exp Hospices/
6 care home*.mp.
7 exp Home Nursing/ or nursing home.mp.
8 exp Terminally Ill/ or terminal illness.mp. or terminally ill.mp. or terminal disease.mp.
9 supportive care.mp.
10 supportive oncology.mp.
11 ((advanced or late-stage or end-stage) and (disease or illness or carcinoma or neoplasm) and (symptom control or pain or cachexia or breathlessness or distress)).mp.
12 (end-stage and (heart or liver or cardiac or kidney or renal)).mp.
13 (dementia and (advanced or late-stage)).mp.
14 1 or 2 or 3 or 4 or 5 or 6 or 7 or 8 or 9 or 10 or 11 or 12 or 13
15 exp ehealth/ or e-health.mp. or e*health.mp.
16 exp Telemedicine/ or tele*medicine.mp.

17 telehealth.mp.
18 (mhealth or m-health or m*health).mp.
19 exp Telephone/ or telephone*.mp.
20 exp Cell Phones/ or cell* phone*.mp. or mobile phone*.mp.
21 (smartphon* or iphone*).mp.
22 (text message* or short messaging service or sms).mp.
23 exp Electronic Health Records/ or electronic health record*.mp. or electronic medical record*.mp.
24 patient health record*.mp.
25 personal health record*.mp.
26 electronic health.mp.
27 (teleconsult* or tele*consult*).mp.
28 telecare.mp.
29 exp Medical Informatics/
30 exp Computer Communication Networks/
31 exp Telecommunications/ or telecommunicat*.mp.
32 (Information and communication technolog*).mp.
33 exp Videoconferencing/ or videoconsult*.mp. or video consult*.mp. or videocommunicat*.mp. or video communicat*.mp. or skype.mp. or facetime.mp.
34 (laptop tablet* or tablet computer* or ipad*).mp.
35 (telemonitor* or ecommuncat*).mp.
36 15 or 16 or 17 or 18 or 19 or 20 or 21 or 22 or 23 or 24 or 25 or 26 or 27 or 28 or 29 or 30 or 31 or 32 or 33 or 34 or 35
37 exp "Review Literature as Topic"/
38 exp "Review"/
39 exp Meta-Analysis/ or exp Meta-Analysis as Topic/
40 (systematic* review* or systematic literature review*).mp.
41 exp Evidence-Based Medicine/
42 evidence synthesis.mp.
43 best practice*.mp.
44 "*systematic*".m_titl.
45 37 or 38 or 39 or 40 or 41 or 42 or 43 or 44
46 14 and 36 and 45
47 limit 46 to yr="2006-Current"

MEDLINE In-Process & Other Non-Indexed Citations

1 palliat*.mp.
2 (end-of-life or end of life).mp.
3 last year of life.mp.
4 terminal care.mp.
5 hospice*.mp.

6 care home*.mp.

7 (home nursing or nursing home*).mp.

8 (terminally ill or terminal illness or terminal disease).mp.

9 supportive care.mp.

10 supportive oncology.mp.

11 ((advanced or late-stage or end-stage) and (disease or illness or carcinoma or neoplasm) and (symptom control or pain or cachexia or breathlessness or distress)).mp.

12 (end-stage and (hear or liver or cardiac or kidney or renal)).mp.

13 (dementia and (advanced or late-stage)).mp.

14 1 or 2 or 3 or 4 or 5 or 6 or 7 or 8 or 9 or 10 or 11 or 12 or 13

15 (ehealth or e-health or e*health).mp.

16 (telemedicine or tele*medicine).mp.

17 telehealth.mp.

18 (mhealth or m-health or m*health).mp.

19 telephone*.mp.

20 (cell phone* or mobile phone*).mp.

21 (smartphon* or iphone*).mp.

22 (text message* or short messaging service or sms).mp.

23 (electronic health record* or electronic medical record*).mp.

24 patient health record*.mp.

25 personal health record*.mp.

26 electronic health.mp.

27 (teleconsult* or tele*consult*).mp.

28 telecare.mp.

29 medical informatics.mp.

30 computer communication networks.mp.

31 telecommunicat*.mp.

32 "information and communication technolog*".mp.

33 (videoconferenc* or videoconsult* or video consult* or videocommunicat* or video communicat* or skype or facetime).mp.

34 (laptop tablet* or tablet computer* or ipad*).mp.

35 (telemonitor* or ecommuncat*).mp.

36 15 or 16 or 17 or 18 or 19 or 20 or 21 or 22 or 23 or 24 or 25 or 26 or 27 or 28 or 29 or 30 or 31 or 32 or 33 or 34 or 35

37 (review* or literature review*).mp.

38 meta-analysis.mp.

39 (systematic* review* or systematic literature review*).mp.

40 evidence-based medicine.mp.

41 evidence synthesis.mp.

42 best practice*.mp.
43 "*systematic*".m_titl.
44 37 or 38 or 39 or 40 or 41 or 42 or 43
45 14 and 36 and 44
46 limit 45 to yr="2006-Current"

PsychINFO

1 palliat*.mp.
2 (end-of-life or end of life).mp.
3 last year of life.mp.
4 terminal care.mp.
5 exp Hospice/ or hospice*.mp.
6 exp Home Care/ or care home.mp.
7 exp Nursing Homes/ or nursing home.mp.
8 exp Terminally Ill Patients/ or exp Terminal Cancer/ or terminal illness.mp. or terminally ill.mp. or terminal disease.mp.
9 supportive care.mp.
10 supportive oncology.mp.
11 ((advanced or late-stage or end-stage) and (disease or illness or carcinoma or neoplasm) and (symptom control or pain or cachexia or breathlessness or distress)).mp.
12 (end-stage and (heart or liver or cardiac or kidney or renal)).mp.
13 (dementia and (advanced or late-stage)).mp.
14 1 or 2 or 3 or 4 or 5 or 6 or 7 or 8 or 9 or 10 or 11 or 12 or 13
15 (ehealth or e-health or e*health).mp.
16 exp Telemedicine/ or tele*medicine.mp.
17 telehealth.mp.
18 (mhealth or m-health or m*health).mp.
19 exp Telephone Systems/ or telephone*.mp.
20 exp Cellular Phones/ or exp Mobile Devices/ or cell* phone*.mp. or mobile phone*.mp.
21 (smartphon* or iphone*).mp.
22 exp Messages/ or text message*.mp. or short messaging service.mp. or sms.mp.
23 (electronic health record* or electronic medical record*).mp.
24 patient health record*.mp.
25 personal health record*.mp.
26 electronic health.mp.
27 (teleconsult* or tele*consult*).mp.
28 telecare.mp.
29 exp computer mediated communication/

30 medical Informatics.mp.
31 computer communication networks.mp.
32 exp Telecommunications Media/ or telecommunicat*.mp.
33 "information and communication technolog*".mp.
34 exp Teleconferencing/ or videoconferenc*.mp. or videoconsult*.mp. or video consult*.mp.
or videocommunicat*.mp. or video communicat*.mp. or skype.mp. or facetime.mp. [mp=title, abstract, heading word, table of contents, key concepts, original title, tests & measures]
35 (laptop tablet* or tablet computer* or ipad*).mp.
36 (telemonitor* or telecommunicat* or ecommuncat*).mp.
37 15 or 16 or 17 or 18 or 19 or 20 or 21 or 22 or 23 or 24 or 25 or 26 or 27 or 28 or 29 or 30 or 31 or 32 or 33 or 34 or 35 or 36
38 exp "Literature Review"/
39 review*.mp.
40 exp Meta Analysis/ or meta-analysis.mp.
41 (systematic* review* or systematic literature review*).mp.
42 exp Evidence Based Practice/ or evidence based medicine.mp.
43 evidence synthesis.mp.
44 exp Best Practices/ or best practice*.mp.
45 "*systematic*".m_titl.
46 38 or 39 or 40 or 41 or 42 or 43 or 44 or 45
47 14 and 37 and 46
48 limit 47 to yr="2006-Current"

Web of Science

#44 "#43 AND #35 AND #13
43 "#42 OR #41 OR #40 OR #39 OR #38 OR #37 OR #36
42 "TITLE: (systematic)
41 "TOPIC: (""best practice*"")
40 "TOPIC: (""evidence-based medicine"")
39 "TOPIC: (""systematic* review*"" OR ""systematic literature review*"")
38 "TOPIC: (""meta-analysis"" OR ""meta analysis"")
37 "TOPIC: (review)
36 "TOPIC: (""literature review"")
35 "#34 OR #33 OR #32 OR #31 OR #30 OR #29 OR #28 OR #27 OR #26 OR #25 OR #24 OR #23 OR #22 OR #21 OR #20 OR #19 OR #18 OR #17 OR #16 OR #15 OR #14
34 "TOPIC: (telemonitor* OR ecommuncat*)
33 "TOPIC: (""laptop tablet*"" OR ""tablet computer*"" OR ipad*)
32 "TS=(videoconferencing OR videoconsult* OR ""video consult*"" OR videocommunicat* OR ""video communicat*"" OR skype OR facetime)

# 31	"TOPIC: (""information and communication technolog*"")
# 30	"TOPIC: (telecommunicat*)
# 29	"TOPIC: (""computer communication network*"")
# 28	"TOPIC: (""medical informatics"")
# 27	"TOPIC: (telecare)
# 26	"TOPIC: (teleconsult* OR tele*consult*)
# 25	"TOPIC: (""electronic health"")
# 24	"TS=(""personal health record*"")
# 23	"TOPIC: (""patient health record*"")
# 22	"TOPIC: (""electronic health record*"" OR ""electronic medical record*"")ag*"" OR ""short messaging service"" or sms)
# 20	"TOPIC: (smartphon* OR iphone*)
# 19	"TOPIC: (""cell* phone*"" OR ""mobile phone"")
# 18	"TOPIC: (telephone)
# 17	"TOPIC: (""mhealth"" or ""m-health"")
# 16	"TOPIC: (telehealth)
# 15	"TOPIC: (telemedicine OR tele*medicine)
# 14	"TS=(""ehealth"" OR ""e-health"")
# 13	"#12 OR #11 OR #10 OR #9 OR #8 OR #7 OR #6 OR #5 OR #4 OR #3 OR #2 OR #1
# 12	"TOPIC: (dementia AND (advanced OR ""late-stage""))
# 11	"TOPIC: (((""end-stage"") AND (heart or liver or cardiac or kidney or renal)))
# 10	"TS=((advanced OR ""late-stage"" OR ""end-stage"") AND (disease OR illness OR carcinoma OR neoplasm) AND (symptom control OR pain OR cachexia OR breathlessness OR distress))
# 9	"TOPIC: (""supportive care"")
# 8	"TOPIC: (""terminally ill"" OR ""terminal illness"" OR ""terminal disease"")
# 7	"TOPIC: (""nursing home*"")
# 6	"TOPIC: (""care home*"")
# 5	"TS=(hospice*)
# 4	"TOPIC: (""terminal care"")
# 3	"TS=(""last year of life"")
# 2	"TS=(""end-of-life"" OR ""end of life"")
# 1	"TS=(palliat*)

CINAHL

S50	S18 AND S40 AND S49
S49	S41 OR S42 OR S43 OR S44 OR S45 OR S46 OR S47 OR S48
S48	TI systematic
S47	"best practice*"
S46	(MH "Meta Synthesis") OR "evidence synthesis"
S45	(MH "Medical Practice, Evidence-Based") OR (MH "Nursing Practice, Evidence-Based") OR "evidence-based medicine"
S44	(MH "Systematic Review") OR "systematic* review*" OR "systematic literature review"

S43 (MH "Meta Analysis") OR "meta-analysis"
S42 "review"
S41 (MH "Literature Review") OR "literature review"
S40 S19 OR S20 OR S21 OR S22 OR S23 OR S24 OR S25 OR S26 OR S27 OR S28 OR S29 OR S30 OR S31 OR S32 OR S33 OR S34 OR S35 OR S36 OR S37 OR S38 OR S39
S39 "telemonitor*" OR "ecommuncat*"
S38 (MH "Computers, Portable") OR "laptop tablet*" OR "tablet computer*" OR "ipad*"
S37 (MH "Videoconferencing") OR "video conferenc*"OR "videoconsult*"OR "video consult*" OR
"videocommunicat*" OR "video communicat*" OR "skype" OR "facetime"
S36 "information and communication technolog*"
S35 (MH "Telecommunications") OR "telecommunicat*"
S34 (MH "Computer Communication Networks") OR "computer communication* network*"
S33 (MH "Health Informatics") OR (MH "Medical Informatics") OR (MH "Nursing Informatics") OR "medical informatics"
S32 "telecare"
S31 ""teleconsult*" OR "tele*consult*""
S30 "electronic health"
S29 (MH "Medical Records, Personal") OR "personal health record*"
S28 "patient health record*"
S27 (MH "Computerized Patient Record") OR "electronic health record*" OR "electronic medical
record*"
S26 (MH "Text Messaging") OR (MH "Instant Messaging") OR "test messag*" OR "short messaging service" OR "sms"
S25 ""smart phone*" or "iphone*""
S24 (MH "Cellular Phone") OR "cell* phone*" OR "mobile phone*"
S23 (MH "Telephone") OR (MH "Telephone Information Services") OR "telephone*"
S22 ""mhealth" OR "m-health""
S21 (MH "Telehealth") OR "telehealth"
S20 (MH "Telemedicine") OR "telemedicine"
S19 ""ehealth" OR "e-health""
S18 S1 OR S2 OR S3 OR S4 OR S5 OR S6 OR S7 OR S8 OR S9 OR S10 OR S11 OR S12 OR S13 OR S14 OR S15 OR S16 OR S17
S17 ""dementia" AND "advanced or late-stage""
S16 ""end-stage" AND "heart" OR liver" OR "cardiac" or "kidney" OR "renal""
S15 "advanced" OR "late-stage" OR "end-stage" AND "disease" OR "illness" OR "carcinoma" OR "neoplasm" AND "symptom control" OR "pain" OR "cachexia" OR "breathlessness" OR "distress"
S14 "supportive oncology"
S13 "supportive care"
S12 ""terminal disease" OR "terminal illness""
S11 (MH "Terminally Ill Patients")
S10 (MH "Home Nursing") OR (MH "Nursing Homes")

S9 "care home*"
S8 "hospice*"
S7 (MH "Hospice and Palliative Nursing") OR (MH "Hospice Patients")
S6 (MH "Hospices") OR (MH "Hospice Care")
S5 (MH "Terminal Care")
S4 ""last year of life""
S3 ""end-of-life" OR "end of life""
S2 (MH "Palliative Care")
S1 "palliat*"

CSDR and DARE

#1 palliat*
#2 MeSH descriptor: [Palliative Care] explode all trees
#3 MeSH descriptor: [Hospice and Palliative Care Nursing] explode all trees
#4 "end-of-life" or "end of life"
#5 "last year of life"
#6 MeSH descriptor: [Terminal Care] explode all trees
#7 MeSH descriptor: [Hospices] explode all trees
#8 "care home*"
#9 MeSH descriptor: [Nursing Homes] explode all trees
#10 MeSH descriptor: [Home Nursing] explode all trees
#11 MeSH descriptor: [Terminally Ill] explode all trees
#12 "terminal illness" or "terminal disease"
#13 "supportive care"
#14 "supportive oncology"
#15 (advanced or "late-stage" or "end-stage") and (disease or illness or carcinoma or neoplasm and "symptom control" or pain or cachexia or breathlessness or distress)
#16 "end-stage" and (heart or liver or cardiac or kidney or renal)
#17 dementia and (advanced or "late-stage")
#18 #1 or #2 or #3 or #4 or #5 or #6 or #7 or #8 or #9 or #10 or #11 or #12 or #13 or #14 or #15 or #16 or #17 29385
#19 ehealth or "e-health"
#20 MeSH descriptor: [Telemedicine] explode all trees
#21 telehealth
#22 mhealth or "m-health"
#23 MeSH descriptor: [Telephone] explode all trees
#24 MeSH descriptor: [Cell Phones] explode all trees
#25 MeSH descriptor: [Smartphone] explode all trees
#26 iphone*
#27 MeSH descriptor: [Text Messaging] explode all trees
#28 MeSH descriptor: [Electronic Health Records] explode all trees

#29 "electronic medical record*"
#30 "patient health record*"
#31 "personal health record*"
#32 "electronic health"
#33 MeSH descriptor: [Remote Consultation] explode all trees
#34 teleconsult* or "tele*consult*"
#35 telecare
#36 MeSH descriptor: [Medical Informatics] explode all trees
#37 MeSH descriptor: [Computer Communication Networks] explode all trees
#38 MeSH descriptor: [Telecommunications] explode all trees
#39 "Information and communication technolog*"
#40 MeSH descriptor: [Videoconferencing] explode all trees
#41 videoconsult* or "video consult*" or videocommunicat* or "video communicat*" or skype or facetime
#42 MeSH descriptor: [Computers, Handheld] explode all trees
#43 "laptop tablet*" or "tablet computer*" or ipad*
#44 telemonitor* or ecommuncat*
#45 #19 or #20 or #21 or #22 or #23 or #24 or #25 or #26 or #27 or #28 or #29 or #30 or #31 or #32 or #33 or #34 or #35 or #36 or #37 or #38 or #39 or #40 or #41 or #42 or #43 or #44
#46 #18 and #45

WHO Global Library Regional Indexes

(tw:(“palliat*” OR “end-of-life” OR “end of life” OR “last year of life” OR “terminal care” OR “hospice*” OR “care home*” OR “home nursing” OR “nursing home*” OR “terminally ill” OR ”terminal illness” OR “terminal disease” OR (“advanced” OR “late-stage“ OR “end-stage“ AND “disease“ OR “illness“ OR “carcinoma“ OR “neoplasm“ AND “symptom control“ OR“ pain“ OR “cachexia“ OR “breathlessness“ OR “distress“) OR (“dementia“ AND “advanced“ OR “late-stage“))) AND (tw:(“ehealth“ OR “e-health“ OR “telemedicine“ OR “telehealth“ OR “mhealth” OR “m-health” OR “telephone” OR “cell* phone*” OR “mobile phone*” OR “smartphon*” OR “iphone*” OR “text message*” OR “short messaging service” OR “sms” OR “electronic health record*” OR “electronic health record*” OR “patient health record*” OR “personal health record*” OR “teleconsult*” OR “tele*consult*” OR “telecare” OR “medical informatics” OR “computer communication networks” OR “telecommunicat*” OR “"information and communication technolog*” OR “videoconferenc*” OR “videoconsult*” OR “video consult*” OR “videocommunicat*” OR “video communicat*” OR “skype” OR “facetime” OR “laptop tablet*” OR ” tablet computer*” OR “ipad*”)) AND (tw:(“review*” OR “literature review” OR “meta-analysis” OR “systematic* review*” OR “systematic literature review*” OR “evidence-based medicine” OR “evidence synthesis” OR “best practice*”))
